# Contribution of Asymptomatic *Plasmodium* Infections to the Transmission of Malaria in Kayin State, Myanmar

**DOI:** 10.1093/infdis/jiy686

**Published:** 2018-11-29

**Authors:** Victor Chaumeau, Ladda Kajeechiwa, Bénédicte Fustec, Jordi Landier, Saw Naw Nyo, Saw Nay Hsel, Phabele Phatharakokordbun, Prapan Kittiphanakun, Suphak Nosten, May Myo Thwin, Saw Win Tun, Jacher Wiladphaingern, Gilles Cottrell, Daniel M Parker, Myo Chit Minh, Nittpha Kwansomboon, Selma Metaane, Céline Montazeau, Kitti Kunjanwong, Sunisa Sawasdichai, Chiara Andolina, Clare Ling, Warat Haohankhunnatham, Peter Christiensen, Sunaree Wanyatip, Kamonchanok Konghahong, Dominique Cerqueira, Mallika Imwong, Arjen M Dondorp, Theeraphap Chareonviriyaphap, Nicholas J White, François H Nosten, Vincent Corbel

**Affiliations:** 1 Centre hospitalier universitaire de Montpellier, Montpellier; 2 UMR 224 “Maladies Infectieuses et Vecteurs, Ecologie, Génétique, Evolution et Contrôle,” Institut de Recherche pour le Développement, Montpellier; 3 Shoklo Malaria Research Unit, Mahidol-Oxford Tropical Medicine Research Unit, Faculty of Tropical Medicine, Mahidol University, Mae Sot; 4 Centre for Tropical Medicine and Global Health, Nuffield Department of Medicine, University of Oxford, United Kingdom; 5 Institut de Recherches pour le Développement, Aix Marseille Univ, INSERM, SESSTIM, Marseille; 6 UMR 216 “Mère et enfant face aux infections tropicales,” Institut de Recherche pour le Développement, Université Paris Descartes, Paris, France; 7 Department of Molecular Tropical Medicine and Genetics, Faculty of Tropical Medicine, Mahidol University; 8 Mahidol-Oxford Tropical Medicine Research Unit, Faculty of Tropical Medicine, Mahidol University; 9 Department of Entomology, Faculty of Agriculture, Kasetsart University, Bangkok, Thailand; 10 Department of Population Health and Disease Prevention, University of California, Irvine

**Keywords:** Mass drug administration, malaria, entomological inoculation rate, primaquine, *Plasmodium falciparum*, *Plasmodium vivax*, elimination, artemisinin resistance, Southeast Asia

## Abstract

**Background:**

The objective of mass antimalarial drug administration (MDA) is to eliminate malaria rapidly by eliminating the asymptomatic malaria parasite reservoirs and interrupting transmission. In the Greater Mekong Subregion, where artemisinin-resistant *Plasmodium falciparum* is now widespread, MDA has been proposed as an elimination accelerator, but the contribution of asymptomatic infections to malaria transmission has been questioned. The impact of MDA on entomological indices has not been characterized previously.

**Methods:**

MDA was conducted in 4 villages in Kayin State (Myanmar). Malaria mosquito vectors were captured 3 months before, during, and 3 months after MDA, and their *Plasmodium* infections were detected by polymerase chain reaction (PCR) analysis. The relationship between the entomological inoculation rate, the malaria prevalence in humans determined by ultrasensitive PCR, and MDA was characterized by generalized estimating equation regression.

**Results:**

Asymptomatic *P. falciparum* and *Plasmodium vivax* infections were cleared by MDA. The *P. vivax* entomological inoculation rate was reduced by 12.5-fold (95% confidence interval [CI], 1.6–100-fold), but the reservoir of asymptomatic *P. vivax* infections was reconstituted within 3 months, presumably because of relapses. This was coincident with a 5.3-fold (95% CI, 4.8–6.0-fold) increase in the vector infection rate.

**Conclusion:**

Asymptomatic infections are a major source of malaria transmission in Southeast Asia.

## Teaser

This study demonstrates the major contribution of asymptomatic low-density *Plasmodium* infections to the transmission of malaria in Southeast Asian settings. Mass drug administration with dihydroartemisinin-piperaquine can interrupt malaria transmission rapidly in areas where the prevalence of asymptomatic infection is high.

Artemisinin resistance in *Plasmodium falciparum* has emerged and spread in the Greater Mekong Subregion [1], leading to the failure of several artemisinin-based combination therapies (ACTs) [2]. Multidrug-resistant parasites spreading from western Cambodia are responsible for a recent resurgence of the disease across the eastern part of the Greater Mekong Subregion [3]. Meanwhile in Myanmar (in the western Greater Mekong Subregion), the incidence of clinical malaria cases has declined [4]. In this area, dihydroartemisinin-piperaquine and artemether-lumefantrine remain effective against *P. falciparum*. It is therefore urgent to eliminate falciparum malaria in Myanmar, the main gateway to India and Bangladesh, before parasites also develop resistance to these 2 ACTs.

Community-wide access to early diagnosis and treatment with an effective ACT is the most effective strategy to reduce the transmission of falciparum malaria [5]. In this region, insecticide-impregnated bed nets have only a marginal effect on the relevant anopheline mosquito vectors [6]. Early diagnosis and treatment limit the transmission that occurs from symptomatic individuals. However, prevalence surveys conducted with ultrasensitive diagnostic tools have revealed that infection with *Plasmodium* parasites is frequently asymptomatic in the Greater Mekong Subregion [7]. Thus, in this area of low endemicity and unstable transmission, healthy residents commonly harbor malaria parasites at low densities, below the detection threshold of microscopy or rapid diagnostic tests [8]. Over time, waves of higher density (although still asymptomatic) parasitemia occur with the sequential emergence of new antigenic variants, generating potentially transmissible densities of gametocytes [9]. Numerous studies have demonstrated the infectivity of low-density *Plasmodium* infections to mosquitoes in areas of low endemicity [10–15]. The probability that a mosquito will become infected when feeding on a human host depends on the prevalence and density of mature-gametocyte carriage [16–19]. Although gametocyte densities are low in asymptomatic carriers, the prevalence of asymptomatic infection is substantially higher than that in individuals with high gametocyte densities (10%–50% vs 0%–2%) [7]. In addition, the duration of infection in asymptomatic carriers is substantially longer than that in symptomatic individuals, who usually seek antimalarial treatment [9, 20]. The contribution of these protracted low-density infections to malaria transmission remains unresolved. According to a recent report from the World Health Organization Evidence Review Group on Low-Density Malaria Infections, “current evidence is insufficient for understanding the contribution of low-density [*Plasmodium*] *falciparum* or [*Plasmodium*] *vivax* infections to onward transmission to human populations. Intervention trials to directly assess the effect of identifying and treating low-density infections are warranted” [21p^16^].

We have shown previously that mass drug administration (MDA) with dihydroartemisinin-piperaquine is effective in clearing the malaria parasite reservoirs in asymptomatic individuals, even in areas with low-level piperaquine resistance among *P. falciparum* parasites [9], and that this effect is sustained over time [22, 23]. However, for *P. vivax* infections, the effect on prevalence was only transient: the asymptomatic reservoir reconstituted within 3 months of the intervention, presumably because of relapses from persistent liver stages of the parasite (ie, hypnozoites) [22]. Primaquine given for 7–14 days is the only drug effective against hypnozoites (radical cure is achieved). However, its routine use is difficult because of the hemolytic risk in patients with glucose-6-phosphate dehydrogenase (G6PD) deficiency. Although rapid diagnostic tests to detect G6PD deficiency are available, they cannot detect deficiency in heterozygous female patients with intermediate G6PD activity, and they have not been distributed widely.

Entomological data characterizing the contribution of asymptomatic carriers to malaria transmission in Southeast Asia are lacking, and there have been no assessments of the impact of MDA on entomological indices. These uncertainties may have contributed to unclear policies for malaria elimination in the Greater Mekong Subregion [21]. The objective of this study was to determine the relationship between asymptomatic malaria parasite reservoirs and corresponding entomological indices and to assess the impact of MDA on malaria transmission.

## MATERIAL AND METHODS

### Study Design

#### Study Sites

The study was conducted in Kayin State, Myanmar, from 2013 to 2015. Four villages, namely A1-KNH, A2-TOT, B1-TPN, and B2-HKT, were selected because they had high prevalences of malaria parasite infection (all species), as determined by high-volume ultrasensitive polymerase chain reaction (uPCR) analysis [22].

#### Intervention

A malaria post was set up in each village to provide community-wide access to early diagnosis and treatment, and long-lasting insecticide-treated bed nets were provided to all villagers. Three rounds of MDA (1 dose of dihydroartemisinin-piperaquine given on 3 consecutive days and 1 low dose of primaquine given on day 1 or day 3) conducted at 1-month intervals eliminated asymptomatic malaria parasite carriage. MDA was administered sequentially, from months 0 to 2 in group A villages (A1-KNH and A2-TOT) and from months 9 to 11 in group B villages (B1-TPN and B2-HKT).

#### Follow-up

**Figure 1.**
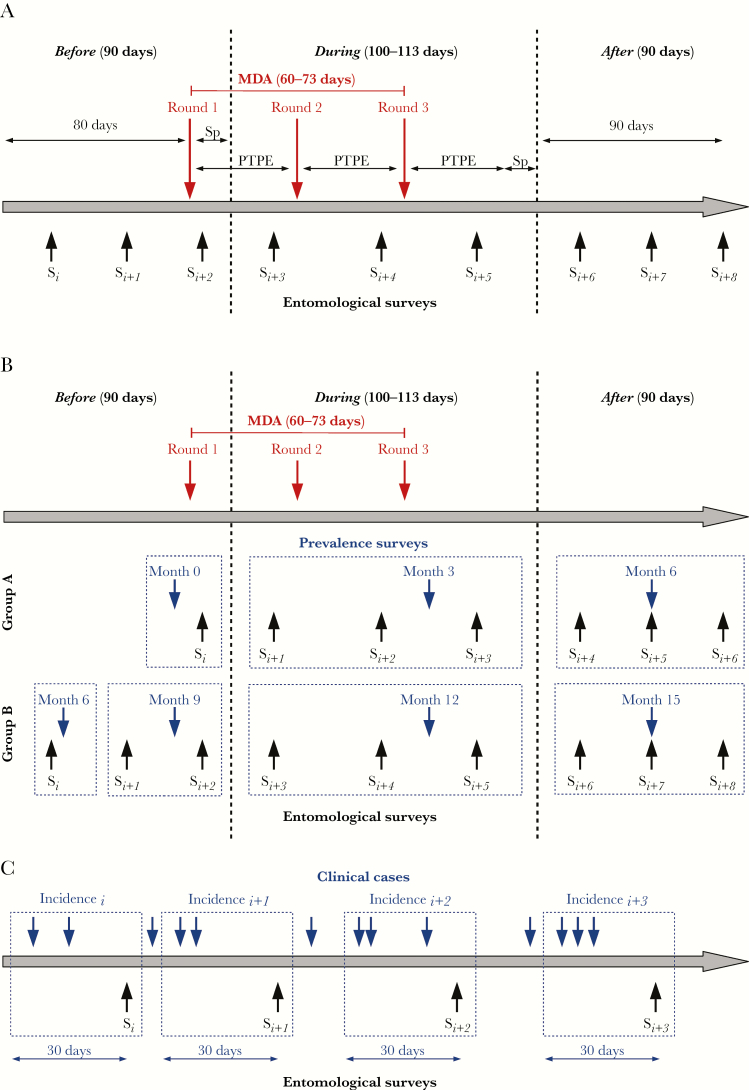
Methods used for the analysis of longitudinal data collected at different time steps in the context of mass antimalarial drug administration. *A*, Matching of the follow-up period with entomological surveys (S*i*: entomological survey at month *i*). The follow-up was divided into 3 categories: before, during, and after MDA campaigns. Entomological surveys potentially impacted by the intervention were assigned to the during-MDA category. The window of MDA impact was defined by taking into account the estimated average durations of *Plasmodium* sporogony in the mosquito vector (Sp; approximately 10 days) and posttreatment prophylactic effects of piperaquine (PTPE; approximately 30 days). Surveys conducted ≥10 days after the beginning of a MDA campaign and ≤40 days after the end of a MDA campaign were assigned to the during-MDA category. Surveys conducted <10 days after the beginning of a MDA campaign and >40 days after the end of a MDA campaign were assigned to before-MDA and after-MDA categories, respectively. *B*, Matching of the cross-sectional prevalence data with entomological surveys. Cross-sectional surveys conducted immediately after the end of a MDA campaign reflect the impact of MDA on malaria prevalence (month 3 for villages A1-KNH and A2-TOT and month 12 for villages B1-TPN and B2-HKT). Entomological surveys assigned to the during-MDA category were matched to the prevalence determined immediately after the end of a MDA campaign. Entomological surveys assigned to the before-MDA and after-MDA categories were matched with the nearest prevalence survey. *C*, Matching of clinical incidence data with entomological surveys. Clinical malaria incidence was aggregated over the month prior to the corresponding entomological survey.

The study period started 80 days before the beginning of MDA campaigns and ended 130 days after they ended (ie, there were 90 days of follow-up before MDA and 90 days of follow-up after MDA). This took into account the approximately 30-day posttreatment prophylactic effect of piperaquine and the approximately 10-day duration of *Plasmodium* sporogony in the anopheline vector [24, 25] (Figure 1). Global positioning system coordinates of households were recorded, and population movement in and out of the villages was monitored through home visits every 2 weeks. Exhaustive cross-sectional surveys were conducted at 3-month intervals, and infection with *Plasmodium* parasites was determined by uPCR (detection threshold, 22 *Plasmodium* genome equivalents/mL blood) [26]. Clinical cases of malaria were diagnosed using the Pf/Pv SD Bioline rapid diagnostic test. *P. falciparum* infections were treated with artemether-lumefantrine and infections with other malaria species were treated with chloroquine, according to standard protocols and Myanmar national policy [27]. Details were recorded at the malaria post. The protocols used to collect mosquitoes and process entomological samples have been described in detail previously [28]. Briefly, entomological surveys were conducted at 1-month intervals in each village, using the indoor and outdoor human-landing catch collection method (there were 50 person-nights of collection per survey). *Anopheles* organisms were identified on the basis of morphologic characteristics, and malaria vectors from the Funestus, Maculatus, and Leucosphyrus groups were analyzed by real-time quantitative PCR, to determine *Plasmodium* infection rates (detection thresholds, 6 and 3.6 sporozoites/mosquito for *P. falciparum* and *P. vivax*, respectively) [29]. The dates of entomological surveys, prevalence surveys, and MDA campaigns are presented in **Supplementary Tables 1–3**. This study is part of a multicenter study conducted in several sites in the Greater Mekong Subregion and is registered at ClinicalTrials.gov (NCT01872702).

### Variables

#### Entomological Indices

The human-biting rate (HBR) was defined as the number of mosquitoes collected, divided by the corresponding number of person-nights of collection. *P. falciparum* and *P. vivax* sporozoite indices (SIs) were defined as the number of mosquitoes testing positive for *P. falciparum* and *P. vivax*, respectively, by PCR, divided by the total number of specimens analyzed. The entomological inoculation rates (EIRs) for *P. falciparum* and *P. vivax* were defined as the number of mosquitoes testing positive for *P. falciparum* and *P. vivax*, respectively, by PCR, divided by the corresponding number of person-nights of collection [30].

#### Timelines

The follow-up period was divided into 3 categories: before, during, and after MDA campaigns. Entomological surveys potentially impacted by the intervention were assigned to the during-MDA category. The window of MDA impact was defined by taking into account the estimated average duration of *Plasmodium* sporogony in the mosquito vector and the posttreatment prophylactic effects of piperaquine. Therefore, entomological surveys conducted ≥10 days after the beginning of a MDA campaign and ≤40 days after the end of a MDA campaign were assigned to the during-MDA category. Entomological surveys conducted <10 days after the beginning of a MDA campaign or >40 days after the end of a MDA campaign were assigned to the before-MDA and after-MDA categories, respectively (Figure 1).

#### Prevalence

Malaria prevalence was defined as the number of individuals positive by uPCR, divided by the total number screened. Prevalence data were aggregated in a 100-m radius around each collection site and matched to entomological data. Cross-sectional surveys conducted immediately after the end of a MDA campaign (ie, at month 3 for villages A1-KNH and A2-TOT and at month 12 for villages B1-TPN and B2-HKT) reflect the impact of MDA on malaria prevalence. Therefore, entomological surveys assigned to the during*-*MDA category were matched to the prevalence determined immediately after the end of a MDA campaign. Entomological surveys assigned to the before-MDA and after-MDA categories were matched with the nearest prevalence survey (Figure 1). The 100-m radius was selected arbitrarily. Values of 60, 160, and 200 m were also tested but did not change the outcome of the model.

#### Incidence

Clinical cases were defined as individuals with fever (aural temperature, ≥37.5°C) or a history of fever in the past 2 days, and malaria parasite infection was confirmed by a rapid diagnostic test or microscopy. The incidence of clinical malaria was calculated for each catching site by aggregating data from all individuals living in households located within a buffer zone (radius, 100 m) around the catching site. For each entomological survey, the incidence was aggregated over the month before the entomological survey (Figure 1). For each entomological survey and each catching site, the incidence was defined as the sum of all clinical cases occurring during the 1-month period in households within the buffer zone, divided by the sum of individual follow-ups accumulated over the 1-month period for persons living in households within the buffer.

#### Season

In this area, the rainy season usually starts in May and ends in November.

### Statistical Analyses

Statistical analyses were performed using R software, version 3.3.2. Exact Poisson confidence intervals (CIs) were estimated for count data (HBR, EIR, and incidence), and exact binomial CIs were estimated for proportions (SI and prevalence). Estimated EIRs were modeled using the generalized estimating equations (GEE) framework with a negative-binomial link to estimate incidence rate ratios (IRRs), an exchangeable correlation structure, and robust standard errors to account for overdispersion and repeated individual-level measurements. HBR, prevalence, and incidence data were divided into quartiles and introduced as categorical variables, to avoid bias from nonlinear relationships between EIR and these covariates. The results of univariable analyses are presented in Supplementary Tables 4 and 5.

### Ethics Approval

This study protocol was reviewed and approved by OxTREC (reference 1017–13 and 1015–13); by the Ethics Review Committee for Research Involving Human Research Subjects, Health Science Group, Chulalongkorn University (COA 154/2014); by the Tak Community Advisory Board; and by village committees. All participants provided their written consent to participate in this study.

## RESULTS

### Baseline Transmission Settings

**Figure 2.**
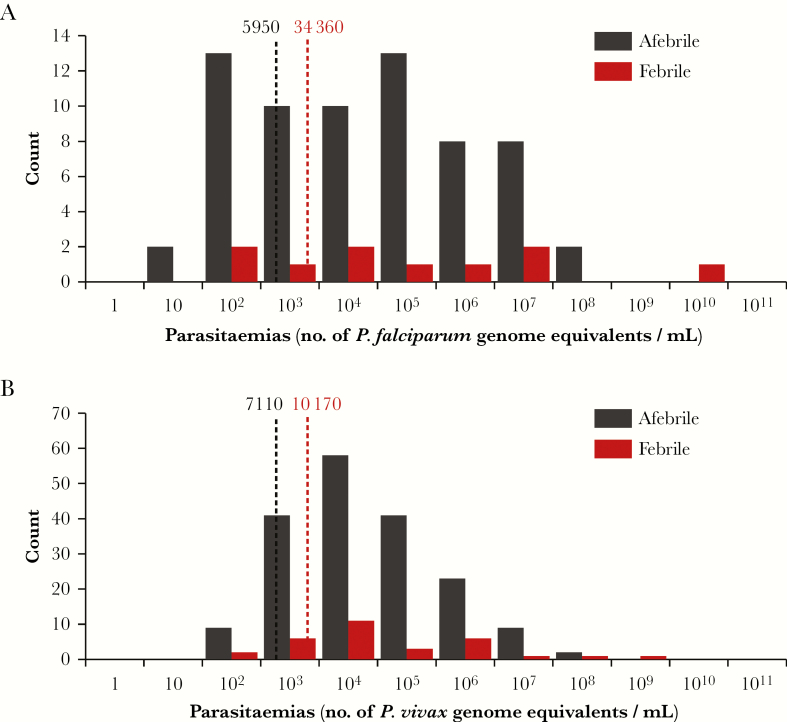
Frequency distribution of malaria parasitemia levels in febrile and afebrile patients in the baseline survey. *A*, *Plasmodium falciparum* infections. *B*, *Plasmodium vivax* infections. Dashed lines indicate geometric mean parasitemia levels.

At baseline, the mean HBR of malaria vectors was 210 bites/person/month (range, 44–241 bites/person/month), and the mean *P. vivax* SI was 2.9 events/1000 mosquitoes (range, 0.0–5.0 events/1000 mosquitoes), yielding an average *P. vivax* EIR of 0.61 infective bites/person/month (range, 0.00–1.21 infective bites/person/month; Table 1). No *P. falciparum*–infected anopheline mosquitoes were identified at baseline, precluding estimations of the *P. falciparum* SI and EIR (Table 2). The baseline incidence of clinical malaria ranged from 0.0 to 1.0 falciparum malaria cases/1000 persons/month and from 6.1 to 8.7 vivax malaria cases/1000 persons/month. The baseline prevalence of *P. falciparum* infection determined by uPCR was high in villages A1-KNH and A2-TOT (23% and 15%, respectively) and low in villages B1-TPN and B2-HKT (0.7% and 1.5%, respectively). In contrast, the baseline prevalence of *P. vivax* infection ranged from 12% in village B1-TPN to 31% in village A2-TOT. Among these infections, individuals in only 15% had an elevated aural temperature (ie, were potentially symptomatic malaria cases). The geometric mean parasitemia levels observed in afebrile and febrile individuals infected with *P. falciparum* were 5950 *Plasmodium* genome equivalents/mL (95% CI, 2110–15 760) and 34 360 *Plasmodium* genome equivalents/mL (95% CI, 990–960 090), respectively. The corresponding figures for *P. vivax* infections were 7110 *Plasmodium* genome equivalents/mL (95% CI, 4570–10 770) and 10 170 *Plasmodium* genome equivalents/mL (95% CI, 2910–38 270), respectively (Figure 2).

**Table 1.** Evolution of the Parasitological and Entomological Indices of Vivax Malaria in the Context of Mass Antimalarial Drug Administration (MAD), Overall and by Village

**Village, Index**	**Before MDA**		**During MDA**		**After MDA**	
	**Raw calculation**	**Value, Mean (95% CI)**	**Raw calculation**	**Value, Mean (95% CI)**	**Raw calculation**	**Value, Mean (95% CI)**
A1-KNH						
Prevalence^a^	52/281	18.5 (14.1–23.5)	2/268	0.7 (.1–2.7)	35/254	13.8 (9.8–18.6)
Incidence^b^	NA	NA	2/1.06	1.89 (.23–6.83)	10/1.03	9.72 (4.66–17.87)
HBR^c^	402/1.67	241 (218; 266)	1352/6.67	203 (192; 214)	742/5	148 (138; 159)
*P. vivax* SI^d^	2/398	5 (0.6; 18)	0/1336	0 (0; 2.8)	5/730	6.8 (2.2; 15.9)
*P. vivax* EIR^e^	2/1.65	1.21 (0.15; 4.38)	0/6.59	0 (0; 0.56)	5/4.92	1.02 (0.33; 2.37)
A2-TOT						
Prevalence^a^	126/410	30.7 (26.3; 35.4)	3/284	1.1 (0.2; 3.1)	32/170	18.8 (13.2; 25.5)
Incidence^b^	NA	NA	4/2.1	1.9 (0.52; 4.87)	9/1.81	4.97 (2.27; 9.43)
HBR^c^	74/1.67	44 (35; 56)	3044/5	609 (587; 631)	2744/5	549 (528; 570)
*P. vivax* SI^d^	0/73	0 (0; 49.3)	2/3012	0.7 (0.1; 2.4)	0/2727	0 (0; 1.4)
*P. vivax* EIR^e^	0/1.64	0 (0; 2.24)	2/4.95	0.4 (0.05; 1.46)	0/4.97	0 (0; 0.74)
B1-TPN						
Prevalence^a^	35/299	11.7 (8.3; 15.9)	2/256	0.8 (0.1; 2.8)	5/225	2.2 (0.7; 5.1)
Incidence^b^	9/1.03	8.7 (3.98; 16.51)	1/0.89	1.12 (0.03; 6.25)	2/0.89	2.25 (0.27; 8.14)
HBR^c^	1521/6.67	228 (217; 240)	353/3.33	106 (95; 118)	618/5	124 (114; 134)
*P. vivax* SI^d^	4/1493	2.7 (0.7; 6.8)	0/352	0 (0; 10.4)	0/609	0 (0; 6)
*P. vivax* EIR^e^	4/6.54	0.61 (0.17; 1.57)	0/3.32	0 (0; 1.11)	0/4.93	0 (0; 0.75)
B2-HKT						
Prevalence^a^	80/453	17.7 (14.3; 21.5)	8/501	1.6 (0.7; 3.1)	53/500	10.6 (8; 13.6)
Incidence^b^	12/2.41	4.99 (2.58; 8.71)	18/2.92	6.16 (3.65; 9.73)	17/2.64	6.44 (3.75; 10.31)
HBR^c^	1511/6.67	227 (215; 238)	3903/5	781 (756; 805)	2103/5	421 (403; 439)
*P. vivax* SI^d^	4/1471	2.7 (0.7; 6.9)	1/3884	0.3 (0; 1.4)	15/2093	7.2 (4; 11.8)
*P. vivax* EIR^e^	4/6.49	0.62 (0.17; 1.58)	1/4.98	0.2 (0.01; 1.12)	15/4.98	3.01 (1.69; 4.97)
Overall						
Prevalence^a^	293/1443	20.3 (18.3; 22.5)	15/1309	1.1 (0.6; 1.9)	125/1149	10.9 (9.1; 12.8)
Incidence^b^	21/3.44	6.1 (3.78; 9.33)	25/6.97	3.59 (2.32; 5.29)	38/6.37	5.97 (4.22; 8.19)
HBR^c^	3508/16.67	210 (204; 218)	8652/20	433 (424; 442)	6207/20	310 (303; 318)
*P. vivax* SI^d^	10/3435	2.9 (1.4; 5.3)	3/8584	0.3 (0.1; 1)	20/6159	3.2 (2; 5)
*P. vivax* EIR^e^	10/16.32	0.61 (0.29; 1.13)	3/19.84	0.15 (0.03; 0.44)	20/19.85	1.01 (0.62; 1.56)

Abbreviations: CI, confidence interval; NA, not applicable; PCR, polymerase chain reaction; *P. vivax*, *Plasmodium vivax*; qPCR, quantitative polymerase chain reaction; uPCR, ultrasensitive polymerase chain reaction.

^a^The prevalence is calculated as the no. of persons positive for *P. vivax* by uPCR */* total no. of persons analyzed. The prevalence is specified as the proportion × 100.

^b^The incidence is calculated as the no. of clinical vivax malaria cases / person-time of follow-up. The incidence is specified as the no. of cases / 1000 persons / mo.

^c^The human-biting rate (HBR) is calculated as the no. of mosquitoes collected (as a proxy for the no. of bites) / corresponding no. of person-nights of collection (only malaria vectors from the Funestus, Maculatus, and Leucosphyrus groups were included in the analysis). The HBR is specified as the no. of bites / person / mo.

^d^The sporozoite index (SI) is calculated as the no. of mosquitoes positive by *Plasmodium* real-time qPCR / total no. of mosquitoes analyzed (only malaria vectors from the Funestus, Maculatus, and Leucosphyrus groups were included in the analysis). The SI is specified as the no. of mosquitoes positive by real-time qPCR / 1000 mosquitoes analyzed.

^e^The entomological inoculation rate (EIR) is calculated the no. of mosquitoes positive by *Plasmodium* real-time qPCR / corresponding no. of person-nights of collection adjusted by the proportion of specimens analyzed by *Plasmodium* real-time qPCR (only malaria vectors from the Funestus, Maculatus, and Leucosphyrus groups were included in the analysis). The EIR is specified as the no. of infective bites / person / mo.

**Table 2.** Evolution of the Parasitological and Entomological Indices of Falciparum Malaria in the Context of Mass Antimalarial Drug Administration, Overall and by Village

**Village, Index**	**Before MDA**		**During MDA**		**After MDA**	
	**Proportion**	**Mean (95% CI)**	**Proportion**	**Mean (95% CI)**	**Proportion**	**Mean (95% CI)**
A1-KNH						
Prevalence^a^	65/281^a^	23.1 (18.3–28.5)	0/268	0 (0–1.4)	2/254	0.8 (.1–2.8)
Incidence^b^	NA^ b^	NA	1/1.06	0.95 (.02–5.27)	2/1.03	1.94 (.24–7.02)
HBR^c^	402/1.67^ c^	241 (218; 266)	1352/6.67	203 (192; 214)	742/5	148 (138; 159)
*P. falciparum* SI^d^	0/398^ d^	0 (0; 9.2)	3/1336	2.2 (0.5; 6.5)	0/730	0 (0; 5)
*P. falciparum* EIR^e^	0/1.65^ e^	0 (0; 2.24)	3/6.59	0.46 (0.09; 1.33)	0/4.92	0 (0; 0.75)
A2-TOT						
Prevalence^a^	62/410	15.1 (11.8; 19)	1/284	0.4 (0; 1.9)	2/170	1.2 (0.1; 4.2)
Incidence^b^	NA	NA	0/2.1	0 (0; 1.76)	2/1.81	1.1 (0.13; 3.99)
HBR^c^	74/1.67	44 (35; 56)	3044/5	609 (587; 631)	2744/5	549 (528; 570)
*P. falciparum* SI^d^	0/73	0 (0; 49.3)	3/3012	1 (0.2; 2.9)	0/2727	0 (0; 1.4)
*P. falciparum* EIR^e^	0/1.64	0 (0; 2.24)	3/4.95	0.61 (0.13; 1.77)	0/4.97	0 (0; 0.74)
B1-TPN						
Prevalence^a^	2/299	0.7 (0.1; 2.4)	0/256	0 (0; 1.4)	2/225	0.9 (0.1; 3.2)
Incidence^b^	1/1.03	0.97 (0.02; 5.38)	0/0.89	0 (0; 4.14)	2/0.89	2.25 (0.27; 8.14)
HBR^c^	1521/6.67	228 (217; 240)	353/3.33	106 (95; 118)	618/5	124 (114; 134)
*P. falciparum* SI^d^	0/1493	0 (0; 2.5)	0/352	0 (0; 10.4)	0/609	0 (0; 6)
*P. falciparum* EIR^e^	0/6.54	0 (0; 0.56)	0/3.32	0 (0; 1.11)	0/4.93	0 (0; 0.75)
B2-HKT						
Prevalence^a^	7/453	1.5 (0.6; 3.2)	0/501	0 (0; 0.7)	1/500	0.2 (0; 1.1)
Incidence^b^	0/2.41	0 (0; 1.53)	0/2.92	0 (0; 1.26)	0/2.64	0 (0; 1.4)
HBR^c^	1511/6.67	227 (215; 238)	3903/5	781 (756; 805)	2103/5	421 (403; 439)
*P. falciparum* SI^d^	0/1471	0 (0; 2.5)	0/3884	0 (0; 0.9)	0/2093	0 (0; 1.8)
*P. falciparum* EIR^e^	0/6.49	0 (0; 0.57)	0/4.98	0 (0; 0.74)	0/4.98	0 (0; 0.74)
Overall						
Prevalence^a^	136/1443	9.4 (8; 11.1)	1/1309	0.1 (0; 0.4)	7/1149	0.6 (0.2; 1.3)
Incidence^b^	1/3.44	0.29 (0.01; 1.62)	1/6.97	0.14 (0; 0.8)	6/6.37	0.94 (0.35; 2.05)
HBR^c^	3508/16.67	210 (204; 218)	8652/20	433 (424; 442)	6207/20	310 (303; 318)
*P. falciparum* SI^d^	0/3435	0 (0; 1.1)	6/8584	0.7 (0.3; 1.5)	0/6159	0 (0; 0.6)
*P. falciparum* EIR^e^	0/16.32	0 (0; 0.23)	6/19.84	0.3 (0.11; 0.66)	0/19.85	0 (0; 0.19)

Abbreviations: CI, confidence interval; NA, not applicable; PCR, polymerase chain reaction; *P. falciparum*, *Plasmodium falciparum*; qPCR, quantitative polymerase chain reaction; uPCR, ultrasensitive polymerase chain reaction.

^a^The proportion is calculated as the no. of persons positive for *P. falciparum* by uPCR */* total no. of persons analyzed. The prevalence is specified as the proportion × 100.

^b^The proportion is calculated as the no. of clinical falciparum malaria cases / person-time of follow-up. The incidence is specified as the no. of cases / 1000 persons / mo.

^c^The proportion is calculated as the no. of mosquitoes collected (as a proxy for the no. of bites) / corresponding no. of person-nights of collection (only malaria vectors from the Funestus, Maculatus, and Leucosphyrus groups were included in the analysis). The human-biting rate (HBR) is specified as the no. of bites / person / mo.

^d^The proportion is calculated as the no. of mosquitoes positive by *Plasmodium* real-time qPCR / total no. of mosquitoes analyzed (only malaria vectors from the Funestus, Maculatus, and Leucosphyrus groups were included in the analysis). The sporozoite index (SI) is specified as the no. of mosquitoes positive by real-time qPCR / 1000 mosquitoes analyzed.

^e^The proportion is calculated the no. of mosquitoes positive by *Plasmodium* real-time qPCR / corresponding no. of person-nights of collection adjusted by the proportion of specimens analyzed by *Plasmodium* real-time qPCR (only malaria vectors from the Funestus, Maculatus, and Leucosphyrus groups were included in the analysis). The entomological inoculation rate (EIR) is specified as the no. of infective bites / person / mo.

### Drivers of Malaria Transmission Intensity

The *P. vivax* EIR was associated positively with the prevalence of asymptomatic malaria and the HBR of mosquito vectors. There was no association between the *P. vivax* EIR and the incidence of symptomatic vivax malaria (Table 3). It was not possible to estimate model coefficients for the *P. falciparum* EIR because there were too few *P. falciparum*–infected mosquitoes in this study. When taking into account the entire 24-month follow-up period described in Chaumeau et al [28] and Landier et al [22], the *P. falciparum* EIR was seasonal and positively associated with the prevalence of mainly asymptomatic malaria and the HBR of mosquito vectors (**Supplementary Table 6**).

**Table 3.** Generalized Estimating Equation Model Output for the Multivariable Analysis of the *Plasmodium vivax* Entomological Inoculation Rate, Including Village, Season, Malaria Vector Human-Biting Rate (HBR), Prevalence, and Incidence Predictors

**Variable, Category**	**IRR (95% CI)**	***P***
Village		
A2-TOT	1 (reference)	
B1-TPN	4.35 (.57–32.91)	.155
A1-KNH	6.59 (1.48–29.32)	.013
B2-HKT	10.82 (2.03–57.53)	.005
Season		
Dry	1 (reference)	
Rainy	2.75 (0.52–14.55)	.233
Prevalence, %^a^		
0–2.5	1 (reference)	
2.5–10	20.45 (2.6–160.51)	.004
10–15	19.11 (4.4–82.95)	<.001
≥ 15	33.15 (4.86–226.19)	<.001
Incidence, cases/1000 persons/mo		
0–1	1 (reference)	
1–10	1.02 (0.1–10.18)	.99
>10	1.29 (0.24–6.91)	.767
HBR, bites/person/mo		
0–60	1 (reference)	
60–160	0.47 (0.04–5.42)	.547
160–350	3.92 (0.91–16.82)	.066
≥ 350	14.62 (2.51–85.1)	.003

Abbreviations: CI, confidence interval; IRR, incidence rate ratio.

^a^By ultrasensitive polymerase chain reaction.

### Impact of MDA on the EIR

The prevalence of asymptomatic falciparum malaria dropped to 0 during MDA and remained below 1% for the 3 months after intervention. Taking into account the 24-month follow-up period, the multivariable GEE model output showed 2-fold and 6-fold reductions in the *P. falciparum* EIR during and after MDA, respectively, but these did not reach statistical significance (**Supplementary Table 7**).

**Figure 3.**
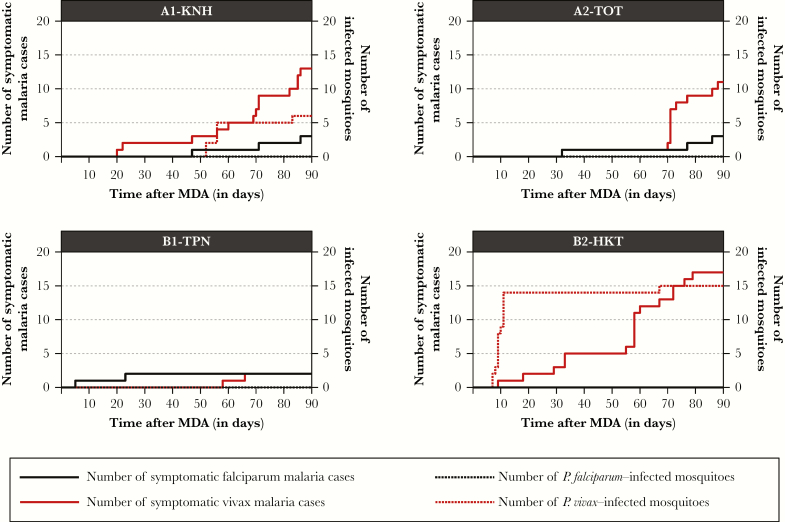
Cumulative number of symptomatic malaria cases and mosquitoes infected with *Plasmodium falciparum* or *Plasmodium vivax* sporozoites after mass antimalarial drug administrations (MDAs), by village. For example, 6 *P*. *vivax*–infected mosquitoes were detected during the 90-day follow-up after an MDA at A1-KNH (2 were detected on 24 November 2013, 3 were detected on 28 November 2013, and 1 was detected on 25 December 2013. The cumulative number of *P. vivax* infected mosquitoes is 2, 5, and 6 on days 52, 56, and 86, respectively.

The prevalence of asymptomatic vivax malaria ranged from 12% to 31% before MDA and dropped to <1.6% in all villages during MDA. Based on the GEE model, MDA was associated with a 12.5-fold decrease (95% CI, 1.6–100) in the *P. vivax* EIR when adjusted for village, season, and HBR covariates (Table 4). The reservoir of asymptomatic vivax infections reconstituted in the 3 months following MDA, presumably because of relapse from hypnozoites. This was coincident with a 5.5-fold increase (95% CI, 4.0–6.3) in *P. vivax*–infected vectors. The prevalence of vivax malaria in village B1-TPN remained lower after MDA intervention when compared to baseline values (2% vs 14%). In this village, the HBR was also lower after MDA than during baseline surveys (124 vs 228 bites/person/months). In all villages, the rise in symptomatic malaria cases after MDA preceded or coincided with the rise in infected vectors, suggesting a causal association (Figure 3). This demonstrates the contribution of asymptomatic parasites reservoirs to malaria transmission and suggests that relapse, rather than external reintroduction of infected vectors, was the main source of reinfection.

**Table 4.** Generalized Estimating Equations Model Output for the Multivariable Analysis of *Plasmodium vivax* Entomological Inoculation Rate, Including Village, Season, Malaria Vector Human-Biting Rate, and Mass Antimalarial Drug Administration (MDA) Predictors

**Variable, Category**	**IRR (95% CI)**	***P***
Village		
A2-TOT	1 (reference)	
B1-TPN	1.77 (.27–11.76)	.553
A1-KNH	3.3 (.42–26.04)	.258
B2-HKT	4.6 (0.64–33.08)	.129
Season		
Dry	1 (reference)	
Rainy	2.98 (0.25–35.15)	.385
MDA intervention		
Before	1 (reference)	
During	0.08 (0.01–0.63)	.016
After	0.42 (0.06–3.01)	.389
HBR, bites/person/mo		
0–60	1 (reference)	
60–160	0.41 (0.03–6.53)	.525
160–350	2.85 (0.44–18.4)	.272
≥ 350	11.8 (1.75–79.36)	.011

Abbreviations: CI, confidence interval; IRR, incidence rate ratio.

## DISCUSSION

In malaria-endemic settings, it is usually not possible to ascribe causal relationships between vector carriage and human infections. An argument against the use of MDA as an elimination accelerator has been that submicroscopic parasite densities do not transmit malaria. This study describes a unique opportunity to assess the contribution of asymptomatic malaria parasite infections to the vectorial transmission of malaria in a low-transmission setting of Southeast Asia. Three rounds of MDA with dihydroartemisinin-piperaquine and a single low dose of primaquine rapidly interrupted malaria transmission in villages where the prevalence of submicroscopic carriage of *Plasmodium* was high. This led to a sustained reduction in the incidence and prevalence of *P. falciparum* malaria but only a transient reduction in the incidence and prevalence of vivax malaria [22]. The timing of the return in *P. vivax* preceding or coinciding with an increase in the prevalence of infected vectors but without concomitant return of *P. falciparum* strongly suggests that relapse, rather than external reintroduction of infected vectors, was the main source of reinfection.

The main limitation of this study is the very low level of endemicity of falciparum malaria, which prevented accurate characterization of *P. falciparum* vector infection. In the study area, the prevalence, incidence, and number of *P. falciparum*–infected mosquitoes were too low to use *P. falciparum* EIR as a primary outcome measure. The intensity of vivax malaria transmission in the study area was much higher than that of falciparum malaria [28], providing sufficient *P. vivax–*infected vectors to assess the contribution of asymptomatic infections to transmission and the impact of MDA. Vantaux et al have suggested that asymptomatic carriage contributes to the transmission of *P. vivax* but not *P. falciparum* [14]. They attributed the lack of infectivity of blood sampled from asymptomatic carriers infected with *P. falciparum* to the low densities of gametocytemia, but the sample was small (60 participants), and the follow-up was only 2 months. In low-transmission settings of Southeast Asia, malaria parasite densities measured by uPCR in asymptomatic individuals fluctuate over 6 orders of magnitude, and carriage may persist for many months [8, 9]. Successive waves of higher asexual parasitemia levels are likely to be followed by peaks of higher transmissibility. The prevalence of gametocyte carriage is high in populations with asymptomatic infections [14], and gametocytes compose a significant proportion of the parasites detected in the blood of asymptomatic individuals [8]. This confirms extensive and detailed prospective studies in volunteers and observations in malaria therapy studies, of the infectiousness to anopheline mosquitoes of asymptomatic *P. falciparum* and *P. vivax* infections [15]. It seems very unlikely therefore that asymptomatic infections with *P. vivax* are transmissible, but not those with *P. falciparum*.

We did not conduct entomological investigations in potential sites of transmission outside the villages (eg, farm huts and forest). In this area, a history of travel outside the village and being a young male are the major risk factors for falciparum malaria [22, 31]. These observations suggest that efficient transmission occurs outside the villages. Malaria vectors in the Thailand-Myanmar borderland belong to the Minimus and Dirus Complexes and to the Maculatus Group [28, 32, 33]. *Anopheles minimus sensu stricto* and *Anopheles maculatus sensu lato* were the most abundant species collected inside the study villages [28]. Highly effective vectors from the Dirus complex are likely to be important contributors to malaria transmission in and around the forest [34] and may have been underestimated in the present study. Somboon et al conducted entomological investigations in Karen villages located in the forest fringe on the Thai side of the Thailand-Myanmar border [32]. They have shown that the biodiversity of *Anopheles* mosquitoes is similar in farm huts located outside the villages and in residential households located inside the villages and that infected malaria vectors could be collected at both sites. In the absence of an animal reservoir of human malaria parasites [35], MDA campaigns probably reduce the transmission cycle of *Plasmodium* outside the village if the coverage of MDA intervention is sufficient.

The carriage of malaria parasites by malaria vectors was associated positively with the prevalence of human malaria parasite infections determined by uPCR. We specifically detected *Plasmodium* sporozoites in the salivary glands, so mosquito infections were relatively old. By multivariable GEE analysis, we estimated that MDA was associated with a 12.5-fold decrease (95% CI, 1.6–100) in the *P. vivax* EIR. The exception was village A2-TOT, in which the response to MDA was much worse than elsewhere and the *P. vivax* EIR increased during MDA. It was not possible to estimate the *P. vivax* EIR accurately during the baseline survey in A2-TOT, because the sample size was small (only 73 malaria vectors were analyzed by *Plasmodium* PCR). MDA participation was poor in this village [22], and the intensity of human-vector contact was higher during and after MDA when compared to that in other villages. These factors probably explained why MDA failed to interrupt malaria in A2-TOT. In another village (B1-TPN), in which the submicroscopic reservoir of *P. vivax* remained low after MDA, the HBR was significantly lower after MDA than before. This suggests that human-vector contact, as well as relapse, contributes to the submicroscopic carriage of *P. vivax*. By contrast, *P. falciparum* does not relapse and so is not expected to rebound following effective MDA campaigns, provided that community-wide access to early diagnosis and treatment prevents reestablishment of local transmission from imported cases.

The impact of MDA on the entomological indices is explained by the pharmacology of the antimalarial drugs used. The posttreatment prophylactic effects of a treatment regimen of piperaquine lasts approximately 30 days [25]. Participants who receive the full 3 rounds of the MDA regimen at 1-month intervals are protected for at least 90 days from reinfection and symptomatic relapse. This 90-day window corresponds to 3–9 generations of malaria parasite vectors, given an estimated longevity of 10–30 days [36–38], during which human-vector transmission of the parasite is also interrupted. Dihydroartemisinin-piperaquine eliminates the asexual stages of all malaria species, as well as *P. vivax* gametocytes and young gametocytes of *P. falciparum* [39], and primaquine kills the mature gametocytes of *P. falciparum,* sterilizing transmissible infections within hours [40, 41]. Mature gametocytes accumulate during submicroscopic infections because of their slower clearance than asexual stages [8, 14], so gametocytocidal doses of primaquine may be more important in asymptomatic carriers than in symptomatic carriers, particularly if a rapid effect is needed. However, in a relatively high-transmission location (Comoros), Deng et al [42] found that addition of a single low dose of primaquine to MDA did not accelerate the decline of symptomatic falciparum malaria. There were no data on submicroscopic carriage.

In low-transmission settings of Southeast Asia, asymptomatic parasite reservoirs contribute to the transmission of malaria and its persistence in affected communities. Three monthly rounds of MDA with dihydroartemisinin-piperaquine and a single low dose of primaquine is an effective intervention that interrupts the transmission cycle of malaria rapidly in these areas, where the prevalence of infection is relatively high and where artemisinin-resistance in *P. falciparum* is established. Without primaquine radical cure, vivax malaria rapidly returns because of relapse.

## Supplementary Data

Supplementary materials are available at *The Journal of Infectious Diseases* online. Consisting of data provided by the authors to benefit the reader, the posted materials are not copyedited and are the sole responsibility of the authors, so questions or comments should be addressed to the corresponding author.

## Supplementary Material

Supplementary Table 1

Supplementary Table 2

Supplementary Table 3

Supplementary Table 4

Supplementary Table 5

Supplementary Table 6

Supplementary Table 7

## Notes

***Acknowledgments.* ** We thank all study participants and communities from the study villages, for their participation and support to the study, and the International Development Cooperation Agency of the Thai Ministry of Foreign Affairs, for their support to the Institut de Recherche pour le Développement–Kasetsart University international research program “STOPVEC.”.

***Financial support.* **This work was supported by the Wellcome Trust (grant 101148; support to the Shoklo Malaria Research Unit, via the Mahidol-Oxford University Research Unit), the Global Fund (grant THA-M-DDC), the Bill and Melinda Gates Foundation (grant GH OPP 1081420), and the Centre Hospitalier Universitaire de Montpellier (support to V. C.).

***Potential conflicts of interest.* **All authors: No reported conflicts.
