# Supplementary material for: Contribution of Asymptomatic Plasmodium Infections to the Transmission of Malaria in Kayin State, Myanmar
Source: J Infect Dis. 2018 Nov 29;219(9):1499–509. doi: 10.1093/infdis/jiy686 (PMC6467188; doi:10.1093/infdis/jiy686)
Supplement: Supplementary Table 1 [file jiy686_suppl_supplementary_table_1.docx]

**Supplementary Table 1.** Dates of the entomological surveys, and matching of entomological surveys with the corresponding exhaustive cross-sectional prevalence surveys and period of follow-up (before, during, and after mass antimalarial drug administration).

| Village | Entomological survey | Start | End | MDA category | Prevalence survey |
| --- | --- | --- | --- | --- | --- |
| A1-KNH | 1 | 11/06/2013 | 15/06/2013 | before | M0 |
| A1-KNH | 2 | 07/07/2013 | 11/07/2013 | during | M3 |
| A1-KNH | 3 | 04/08/2013 | 08/08/2013 | during | M3 |
| A1-KNH | 4 | 01/09/2013 | 05/09/2013 | during | M3 |
| A1-KNH | 5 | 29/09/2013 | 03/10/2013 | during | M3 |
| A1-KNH | 6 | 27/10/2013 | 31/10/2013 | after | M6 |
| A1-KNH | 7 | 24/11/2013 | 28/11/2013 | after | M6 |
| A1-KNH | 8 | 22/12/2013 | 26/12/2013 | after | M6 |
| A2-TOT | 1 | 19/05/2013 | 23/05/2013 | before | M0 |
| A2-TOT | 2 | 14/07/2013 | 18/07/2013 | during | M3 |
| A2-TOT | 3 | 11/08/2013 | 15/08/2013 | during | M3 |
| A2-TOT | 4 | 08/09/2013 | 12/09/2013 | during | M3 |
| A2-TOT | 5 | 06/10/2013 | 10/10/2013 | after | M6 |
| A2-TOT | 6 | 03/11/2013 | 07/11/2013 | after | M6 |
| A2-TOT | 7 | 01/12/2013 | 05/12/2013 | after | M6 |
| B1-TPN | 7 | 10/11/2013 | 14/11/2013 | before | M6 |
| B1-TPN | 8 | 08/12/2013 | 12/12/2013 | before | M6 |
| B1-TPN | 9 | 05/01/2014 | 09/01/2014 | before | M9 |
| B1-TPN | 10 | 02/02/2014 | 06/02/2014 | before | M9 |
| B1-TPN | 11 | 30/03/2014 | 03/04/2014 | during | M12 |
| B1-TPN | 12 | 27/04/2014 | 01/05/2014 | during | M12 |
| B1-TPN | 13 | 25/05/2014 | 29/05/2014 | after | M15 |
| B1-TPN | 14 | 22/06/2014 | 26/06/2014 | after | M15 |
| B1-TPN | 15 | 20/07/2014 | 24/07/2014 | after | M15 |
| B2-HKT | 8 | 12/01/2014 | 16/01/2014 | before | M6 |
| B2-HKT | 9 | 09/02/2014 | 13/02/2014 | before | M9 |
| B2-HKT | 10 | 09/03/2014 | 13/03/2014 | before | M9 |
| B2-HKT | 11 | 06/04/2014 | 10/04/2014 | before | M9 |
| B2-HKT | 12 | 04/05/2014 | 08/05/2014 | during | M12 |
| B2-HKT | 13 | 01/06/2014 | 05/06/2014 | during | M12 |
| B2-HKT | 14 | 29/06/2014 | 03/07/2014 | during | M12 |
| B2-HKT | 15 | 27/07/2014 | 31/07/2014 | after | M15 |
| B2-HKT | 16 | 24/08/2014 | 28/08/2014 | after | M15 |
| B2-HKT | 17 | 21/09/2014 | 25/09/2014 | after | M15 |
