# Supplementary material for: Contribution of Asymptomatic Plasmodium Infections to the Transmission of Malaria in Kayin State, Myanmar
Source: J Infect Dis. 2018 Nov 29;219(9):1499–509. doi: 10.1093/infdis/jiy686 (PMC6467188; doi:10.1093/infdis/jiy686)
Supplement: Supplementary Table 2 [file jiy686_suppl_supplementary_table_2.docx]

**Supplementary Table 2.** Dates of the exhaustive cross-sectional prevalence surveys.

| Village | Survey | Star | End |
| --- | --- | --- | --- |
| A1-KNH | M0 | 12/06/2013 | 14/06/2013 |
| A1-KNH | M3 | 12/09/2013 | 14/09/2013 |
| A1-KNH | M6 | 11/12/2013 | 12/12/2013 |
| A2-TOT | M0 | 27/05/2013 | 31/05/2013 |
| A2-TOT | M3 | 02/09/2013 | 06/09/2013 |
| A2-TOT | M6 | 26/11/2013 | 29/11/2013 |
| B1-TPN | M6 | 07/11/2013 | 08/11/2013 |
| B1-TPN | M9 | 28/01/2014 | 31/01/2014 |
| B1-TPN | M12 | 23/04/2014 | 24/04/2014 |
| B1-TPN | M15 | 14/07/2014 | 15/07/2014 |
| B2-HKT | M6 | 08/01/2014 | 11/01/2014 |
| B2-HKT | M9 | 01/04/2014 | 06/04/2014 |
| B2-HKT | M12 | 24/06/2014 | 27/06/2014 |
| B2-HKT | M15 | 16/09/2014 | 19/09/2014 |
