# Supplementary material for: Contribution of Asymptomatic Plasmodium Infections to the Transmission of Malaria in Kayin State, Myanmar
Source: J Infect Dis. 2018 Nov 29;219(9):1499–509. doi: 10.1093/infdis/jiy686 (PMC6467188; doi:10.1093/infdis/jiy686)
Supplement: Supplementary Table 3 [file jiy686_suppl_supplementary_table_3.docx]

**Supplementary Table 3.** Dates of the mass antimalarial drug administration campaigns.

| Village | Start | End |
| --- | --- | --- |
| A1-KNH | 12/06/2013 | 24/08/2013 |
| A2-TOT | 27/05/2013 | 07/08/2013 |
| B1-TPN | 28/01/2014 | 29/03/2014 |
| B2-HKT | 01/04/2014 | 10/06/2014 |
