# Supplementary material for: Contribution of Asymptomatic Plasmodium Infections to the Transmission of Malaria in Kayin State, Myanmar
Source: J Infect Dis. 2018 Nov 29;219(9):1499–509. doi: 10.1093/infdis/jiy686 (PMC6467188; doi:10.1093/infdis/jiy686)
Supplement: Supplementary Table 4 [file jiy686_suppl_supplementary_table_4.docx]

**Supplementary Table 4.** Generalised estimating equations model output for the univariable analysis of *P. vivax* entomological inoculation rate including village, season, malaria vectors human-biting rate, prevalence, incidence and mass antimalarial drug administration predictors.

| Variable | Category | IRR | 95%CI | p-value |
| --- | --- | --- | --- | --- |
| Village | A2-TOT | 1 | reference | - |
|  | B1-TPN | 1.62 | 0.24 - 11.07 | 0.622 |
|  | A2-KNH | 3.05 | 0.37 - 24.93 | 0.299 |
|  | B2-HKT | 6.94 | 1.04 - 46.12 | 0.045 |
| Season | dry | 1 | reference | - |
|  | rainy | 2.1 | 0.55 - 7.96 | 0.276 |
| HBR | 0 – 60 | 0 | reference | - |
| (bites/person/month) | 60 - 160 | 0.61 | 0.05 - 7.05 | 0.689 |
|  | 160 - 350 | 5.54 | 0.81 - 37.81 | 0.081 |
|  | >350 | 13.53 | 1.89 - 96.68 | 0.009 |
| Prevalence | 0 – 2.5 | 1 | reference | - |
| (%) | 2.5 – 10 | 13.71 | 1.29 - 146.16 | 0.03 |
|  | 10 – 15 | 26.56 | 6.21 - 113.49 | <0.001 |
|  | >15 | 11.86 | 1.05 - 133.53 | 0.045 |
| Incidence | 0 - 1 | 1 | reference | - |
| (cases / 1000 person / month) | 1 - 10 | 0.78 | 0.09 - 6.56 | 0.818 |
|  | >10 | 0.43 | 0.09 - 1.97 | 0.276 |
| MDA intervention | before | 1 | reference | - |
|  | during | 0.25 | 0.05 - 1.27 | 0.094 |
|  | after | 1.66 | 0.48 - 5.8 | 0.424 |
