# Supplementary material for: Contribution of Asymptomatic Plasmodium Infections to the Transmission of Malaria in Kayin State, Myanmar
Source: J Infect Dis. 2018 Nov 29;219(9):1499–509. doi: 10.1093/infdis/jiy686 (PMC6467188; doi:10.1093/infdis/jiy686)
Supplement: Supplementary Table 5 [file jiy686_suppl_supplementary_table_5.docx]

**Supplementary Table 5.** Generalised estimating equations model output for the univariable analysis of *P. falciparum* entomological inoculation rate including village, season, malaria vectors human-biting rate, prevalence, incidence and mass antimalarial drug administration predictors (24-month follow-up described in Landier *et al.* [11] and Chaumeau *et al.* [27]).

| Variable | Category | IRR | 95%CI | p-value |
| --- | --- | --- | --- | --- |
| Village | B1-TPN | 1 | reference | - |
|  | A1-KNH | 1.18 | 0.40 – 3.45 | 0.767 |
|  | B2-HKT | 1.50 | 0.45 – 4.97 | 0.503 |
|  | A2-TOT | 1.76 | 0.58 – 5.29 | 0.316 |
| Season | dry | 1 | reference | - |
|  | rainy | 7.48 | 0.95 - 58.97 | 0.056 |
| HBR | 0 – 60 | 1 | reference | - |
| (bites/person/month) | 60 - 160 | 0.03 | 0.01 - 0.14 | <0.001 |
|  | 160 - 350 | 3.37 | 0.29 – 38.73 | 0.330 |
|  | >350 | 15.88 | 1.90 - 132.62 | 0.011 |
| Prevalence | 0 – 1.0 | 1 | reference | - |
| (%) | 1.0 – 2.5 | 0.91 | 0.16 - 5.1 | 0.915 |
|  | 2.5 – 5 | 1.55 | 0.32 - 7.47 | 0.586 |
|  | >5 | 5.7 | 1.79 - 18.18 | 0.003 |
| Incidence | 0 - 1 | 1 | reference | - |
| (cases / 1000 person / month) | 1 - 15 | 1.84 | 0.34 - 10.09 | 0.48 |
|  | >15 | 3.47 | 0.82 - 14.62 | 0.09 |
| MDA intervention | before | 1 | reference | - |
|  | during | 1.65 | 0.39 - 7.06 | 0.497 |
|  | after | 0.37 | 0.1 - 1.36 | 0.132 |
