# Supplementary material for: Contribution of Asymptomatic Plasmodium Infections to the Transmission of Malaria in Kayin State, Myanmar
Source: J Infect Dis. 2018 Nov 29;219(9):1499–509. doi: 10.1093/infdis/jiy686 (PMC6467188; doi:10.1093/infdis/jiy686)
Supplement: Supplementary Table 6 [file jiy686_suppl_supplementary_table_6.docx]

**Supplementary Table 6.** Generalised estimating equations model output for the multivariable analysis of *P. falciparum* entomological inoculation rate including village, season, malaria vector human-biting rate, prevalence determined by uPCR and incidence predictors (data from the 24-month follow-up described in Landier *et al.* [11] and Chaumeau *et al.* [27]).

| Variable | Category | IRR | 95%CI | p-value |
| --- | --- | --- | --- | --- |
| Village | A2-TOT | 1 | reference | - |
|  | B2-HKT | 1.13 | 0.24 - 5.33 | 0.879 |
|  | A1-KNH | 1.86 | 0.33 - 10.39 | 0.481 |
|  | B1-TPN | 2.85 | 0.5 - 16.33 | 0.241 |
| Season | dry | 1 | reference | - |
|  | rainy | 5.16 | 0.6 - 44.09 | 0.134 |
| HBR | 0 – 60 | 1 | reference | - |
| (bites/person/month) | 60 - 160 | 0.03 | 0 - 0.16 | 0 |
|  | 160 - 350 | 2.94 | 0.19 - 44.39 | 0.437 |
|  | >350 | 15.76 | 2.36 - 105.13 | 0.004 |
| Prevalence | 0 - 1 | 1 | reference | - |
| (in %) | 1 – 2.5 | 1.18 | 0.12 - 11.64 | 0.885 |
|  | 2.5 - 5 | 2.17 | 0.42 - 11.19 | 0.355 |
|  | >5 | 4.59 | 0.78 - 26.89 | 0.091 |
| Incidence | 0 - 1 | 1 | reference | - |
| (cases / 1000 person / month) | 1 - 15 | 2.07 | 0.35 - 12.31 | 0.422 |
|  | >15 | 0.92 | 0.17 - 5.05 | 0.927 |
