# Supplementary material for: Contribution of Asymptomatic Plasmodium Infections to the Transmission of Malaria in Kayin State, Myanmar
Source: J Infect Dis. 2018 Nov 29;219(9):1499–509. doi: 10.1093/infdis/jiy686 (PMC6467188; doi:10.1093/infdis/jiy686)
Supplement: Supplementary Table 7 [file jiy686_suppl_supplementary_table_7.docx]

**Supplementary Table 7.** Generalised estimating equations model output for the multivariable analysis of *P. falciparum* entomological inoculation rate including village; season, malaria vector human-biting rate and mass antimalarial drug administration predictor (data from the 24-month follow-up described in Landier *et al.* [11] and Chaumeau *et al.* [27]).

| Variable | Category | IRR | 95%CI | p-value |
| --- | --- | --- | --- | --- |
| Village | B2-HKT | 1 | reference | - |
|  | B1-TPN | 1.4 | 0.19 - 10.06 | 0.738 |
|  | A2-TOT | 1.6 | 0.56 - 4.57 | 0.378 |
|  | A1-KNH | 1.83 | 0.64 - 5.26 | 0.263 |
| Season | dry | 1 | reference | - |
|  | rainy | 4.74 | 0.55 - 41 | 0.158 |
| HBR | 0 – 60 | 1 | reference | - |
| (bites/person/month) | 60 - 160 | 0.01 | 0 - 0.09 | 0 |
|  | 160 - 350 | 2.47 | 0.15 - 41.12 | 0.53 |
|  | >350 | 12.85 | 1.09 - 151.18 | 0.042 |
| MDA intervention | before | 1 | reference | - |
|  | during | 0.53 | 0.06 - 4.85 | 0.572 |
|  | after | 0.17 | 0.02 - 1.43 | 0.102 |
